# Acetone Fraction of the Red Marine Alga *Laurencia papillosa* Reduces the Expression of Bcl-2 Anti-apoptotic Marker and Flotillin-2 Lipid Raft Marker in MCF-7 Breast Cancer Cells

**DOI:** 10.22037/ijpr.2020.1100933

**Published:** 2020

**Authors:** Josiane Elia, Karina Petit, Jean-Michel Huvelin, Mona Tannoury, Mona Diab-Assaf, Delphine Carbonnelle, Hassan Nazih

**Affiliations:** a *MMS-EA 2160, Department of Biochemistry and Pharmacognosy, Faculty of Pharmacy, University of Nantes, Nantes, France. *; b *Department of Biology, Faculty of Sciences II, Lebanese University, Fanar, Lebanon. *; c *Department of Biochemistry and Chemistry, Faculty of Sciences II, Lebanese University, Fanar, Lebanon.*

**Keywords:** Laurencia papillosa, MCF-7 cells, Cytotoxic activity, Apoptosis, Flotillin-2

## Abstract

Marine macroalgae have attracted much attention in recent years as a valuable source of bioactive metabolites. The cytotoxic potential of the *Laurencia papillosa* red alga collected from the Lebanese coast has been investigated on human breast cancer cells MCF-7. The crude extract of *Laurencia papillosa* (*L. papillosa*) was fractionated by column chromatography using a series of increasingly polar solvents (methylene chloride, acetone and methanol). Cytotoxicity of the crude extract and fractions was determined by MTT assay in MCF-7 cells. Apoptosis was detected by annexin V/propidium iodide assay and by measurement of Bcl-2 expression. Flotillin-2 expression was examined using RT-qPCR and Western blot. The crude extract, and the fractions of CH2Cl2 and acetone exhibited a dose-dependent cytotoxic effect on MCF-7 cells. Apoptosis was specifically induced by one of the acetone fractions having the highest cytotoxicity. It has been demonstrated by an increase in late phase apoptotic cell populations, and a decrease in Bcl-2 anti-apoptotic marker expression on mRNA and protein levels in a dose- and time- dependent manner. Furthermore, this active fraction decreased Flotillin-2 expression associated with cancer progression. Our data suggest that *L. papillosa* is an important source of cytotoxic metabolites. Further studies are needed for the chemical characterization of the metabolite associated with observed biological activities.

## Introduction

Breast cancer is the second most common cancer in women worldwide with about 1.67 million new cancer cases diagnosed in 2012 and the fifth leading cause of cancer death with about 522 000 deaths (1). However, most of the drugs currently used to treat cancer have serious side effects on normal cells, hence the need to develop new effective anticancer agents.

In this context, great attention has been paid to natural products obtained from plants, micro-organisms and marine organisms as a source of anticancer agents. An exhaustive review pointed out that of all new anticancer drugs approved worldwide during the period 1940-2014, 65% were of natural origin and 19% of synthetic origin (2). The different categories of natural origin are either an unaltered natural product, a derivative of a natural product resulting from a semi-synthetic modification, or a product made by total synthesis but the pharmacophore originates from the natural product. Among the drugs from natural sources in clinical use are some examples: vinblastine and paclitaxel derived from plants, actinomycin D and bleomycin A2 derived from microorganisms, cytarabine and trabectedin of marine origin (3, 4).

Covering 70% of the earth’s surface, the oceans represent the largest reservoir of bioactive compounds and are therefore a remarkable resource for the discovery of new pharmaceutical drugs (5). Several anticancer compounds of marine origin have already been approved or are in clinical trials (6, 7).

Marine macroalgae are one of the natural resources of the marine environment that have long been used as a source of functional foods and in traditional medicines (8). The literature has established that marine algae represents a rich source of natural products with unique biological activity and original chemical structure that may be useful in the search for effective and specific drugs for the treatment of human diseases (9-11). In the field of cancer, the potential of marine algae as a source of anticancer drugs has been mainly studied using crude extracts or partially purified fractions (12).

In particular, red macroalgae have been found to be among the main producers of secondary metabolites in the marine environment, the genus *Laurencia* being the most prolific (13). This genus belongs to the Ceramiales order and the Rhodomelaceae family and comprises 146 taxonomically accepted species distributed throughout the world, mainly in tropical and subtropical waters (14). Although *Laurencia* has been widely studied over the past fifty years, many new metabolites are still being reported until today (5, 15 and 16). These metabolites belong to the chemical classes of sesquiterpenes, C15 acetogenins, diterpenes, triterpenes, indoles, steroids, aromatic comp-ounds, and miscellaneous metabolites, most being halogenated (13). Many of these metabolites have been reported to possess various biological activities, such as cytotoxic, antib-acterial, antifungal, antiviral, anti-parasitic, and anti-inflammatory activities.

Lebanon has a large number of marine organisms, but interest in the valorization of marine macroalgae is considered recent compared to other countries. *Laurencia papillosa* (C. Agardh) is widely distributed on the Lebanese rocky coast. In fact, several studies have been conducted on this species collected all over the world (Japan, Red Sea, Caribbean Sea, India), and many metabolites have been isolated, identified, and tested for their biological activity (17-23). Recently, it has been reported that both crude extracts of ethanol/water (50/50, v/v) and ethanol/chloroform (50/50, v/v) of *L. papillosa* collected from the Lebanese coast exhibited moderate cytotoxic activity against Jurkat human cancer cell line (acute T-cell leukemia) with an IC50 of 121.6 and 57.7 μg/mL respectively (24).

This prompted us to conduct further studies on the cytotoxic activity of *Laurencia papillosa* collected from the Lebanese coast on the human breast cancer cell line MCF-7, followed by apoptosis assays.

## Experimental

*Algal material*

Alga sample was collected from Berbara coast in Lebanon, May 2015. Identification was carried out by Dr. Tannoury according to Guiry and Guiry (25). An herbarium voucher specimen No. 700 was deposited in the Herbarium of the Department of Biology, Faculty of Science II, Lebanese University (24). Fresh material was washed successively with tap water and distilled water to remove salt, sand and epiphytes, and then lyophilized and ground very finely.

*Extraction and fractionation*

Lyophilized alga (84 g) was macerated thrice by methylene chloride/methanol (CH2Cl2/MeOH 50/50, v/v) at room temperature (26, 27). The combined extracts were concentrated under reduced pressure at 40 °C using a rotary evaporator. Crude extract (2 g) was then partitioned between methylene chloride and distilled water (73/27, v/v) for desalting. Organic layer was concentrated to dryness to yield the CH2Cl2/MeOH extract (239.5 mg). A small amount (19.8 mg) was then subjected to silica gel column chromatography with successive elution of solvents of increasing polarity (methylene chloride, acetone and methanol) (28).

*Biological and chemical materials*

MCF-7 human breast cancer cells were obtained from the European Collection of Animal Cell Cultures (Salisbury, United Kingdom). Dulbecco’s Modified Eagle’s Medium (DMEM), 3-(4,5-Dimethylthiazol-2-yl)-2,5-Diphenyltetrazolium Bromide (MTT), Dimethyl sulfoxide (DMSO), primers for qPCR, and rabbit antibody against glyceraldehyde-3-phosphate dehydrogenase (GADPH), Bcl-2, and human Flottilin-2 (FLOT2) were purchased from Sigma Aldrich (Saint-Quentin Fallavier, France). Annexin V/propidium iodide (PI) kit, M-PER™ Mammalian Protein Extraction Reagent, and Halt™ Protease and Phosphatase Inhibitor Single-Use Cocktail were purchased from Life Technologies (Saint-Aubin, France). TRIzol reagent for RNA isolation was from Invitrogen (Cergy-Pontoise, France). iScript^TM^ Reverse Transcription Supermix for RT-qPCR and iQ^TM^ SYBR Green Supermix were purchased from Bio-Rad (Marnes-la-Coquette, France). IRDye whole IgG secondary antibodies were from LI-COR Biosciences (Bad Homburg, Germany).

*Cell culture and treatment*

MCF-7 cancer cells were cultured in DMEM supplemented with 10% fetal bovine serum, 1% glutamine and 1% penicillin-streptomycin at 37 °C under a controlled atmosphere of 5% CO2 and 95% humidity. The cells were cultured for 24 h in quadruplicate in 96-well plates (10^4^ cells per well; MTT assay) or in 6-well plates (5 × 10^5^ cells per well; Annexin V-FITC/PI assay, RT-qPCR and Western blot), and then treated with various concentrations of the algal samples which were first dissolved in DMSO at 0.2% and then diluted in DMEM containing 0.1% bovine serum albumin. DMSO at 0.2% was tested as a negative control. Subsequently, cells were incubated for 24, 48, or 72 h at 37 °C.

*MTT cytotoxicity assay*

As described by Carbonnelle *et al*., after the desired incubation time, 50 μL of MTT solution (2.5 mg/mL) were added to each well and incubated for a further 4 h at 37 °C (29). Thereafter, culture medium was removed and 200 μL DMSO were added to each well to solubilize the formazan crystals. A SpectraMax 190 microplate reader was used to measure the absorbance at 570 nm. The samples with an IC50 less than 30 µg/mL were regarded as active and could be considered for further investigation.

*Annexin V-FITC/ propidium iodide assay*

The cells were trypsinized, washed with phosphate-buffered saline, suspended with 1X annexin binding buffer, incubated with Annexin V and PI staining solution for 15 min at room temperature in the dark, and finally analyzed using the Accuri C6 flow cytometer (30).

*RNA extraction and RT-qPCR analysis*

Total RNA was extracted by the TriZol Reagent. The mRNA (1 μg/μL) was then reverse-transcribed into complementary DNA using iScript Reverse Transcription Supermix. A priming step for 5 min at 25 °C was followed by a reverse transcription phase of 30 min at 42 °C, then a reverse transcription inactivation of 5 min at 85 °C. Quantitative PCR was then performed on a MyiQ Real Time PCR Detection System, using the SYBR Green Supermix. PCR was carried out for 45 cycles of 95 °C for 30 s and 60 °C for 30 s (30). Transcript levels of Bcl-2, FLOT2 and the reference gene 18S were determined. The relative quantification was performed using the 2^ΔΔCT^ method, with ΔΔCT = ΔCT reference gene - ΔCT gene of interest. The primer sequences are shown in Table 1.

*Western blot analysis*

Protein extracts were prepared by cell lysis with a buffer composed of M-PER, protease and phosphatase inhibitors, and sodium chloride. Protein concentration was then determined according to the bicinchoninic acid assay using a bovine serum albumin standard. After separating by sodium dodecyl sulfate polyacrylamide gel electrophoresis, proteins (20 µg) were transferred to a nitrocellulose membrane which was then blocked with 5% skim milk at room temperature for 2 h and incubated overnight at 4 °C with primary antibodies against Bcl-2, FLOT2, and GAPDH (a loading control). The membrane was then washed and incubated with secondary antibodies at room temperature. Finally, the proteins bands were visualized and quantified using the Odyssey system (29).

*Data analysis*

The values represent the mean ± standard deviation of three different experiments. Student’s *t*-test was used and *p*-value < 0.05 (*) was considered significantly different from control, *p* < 0.01 (**) moderately significant and *p* < 0.001 (***) highly significant. The IC50 indicating the concentration of the algal sample inhibiting 50% of the cell growth was estimated graphically using the Excel 2007 software.

## Results

*Growth inhibition of MCF-7 cells by *Laur-encia papillosa* crude extract and fractions*

**Figure 1:**
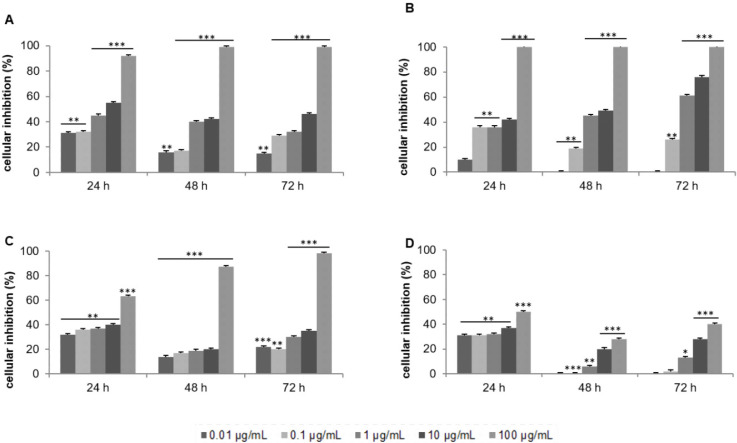
Effect of the crude extract of *Laurencia papillosa* and its fractions on the growth of MCF-7 cells. Cells were cultured for 24 h and treated with the indicated concentrations for 24, 48 and 72 h. Cytotoxicity was determined by MTT assay. (A) CH2Cl2/MeOH extract, (B) CH2Cl2 fractions, (C) acetone fractions, (D) MeOH fractions

The CH2Cl2/MeOH extract exerted a cytotoxic effect in a dose-dependent manner and presented an IC50 of 11.8 μg/mL after 72 h of treatment (Figure 1A). Silica gel column chromatography led to 24 fractions of increasing polarity: F1 to F8 with CH2Cl2, F9 to F16 with acetone and F17 to F24 with MeOH. IC50 was calculated after 72 h of treatment for each solvent. Despite a modest cytotoxic activity of the acetone fraction (IC50 of 17.3 μg/mL; Figure 1C) compared to that of CH2Cl2 fraction (IC50 of 0.4 μg/mL; Figure 1B), the dose-response curves were more consistent with the potential future therapeutic use of the acetone fraction. 

**Figure 2:**
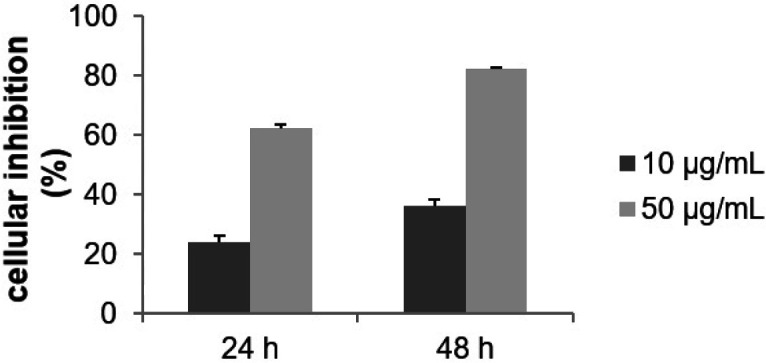
Effect of F9 acetone fraction on the growth of MCF-7 cells. Cells were treated with the indicated concentration for 24 and 48 h. Cytotoxicity was evaluated by MTT assay

Based on our criteria, the MeOH fraction was inactive (IC50 > 100 μg/mL; Figure 1D). The cytotoxic activity on the MCF-7 cells of each acetone fractions was then evaluated at 10 and 50 μg/mL for 24 and 48 h. F10 to F16 were inactive, the IC50 was greater than 50 μg/mL after 48 h of incubation (data not shown). The comprehensive investigation thus highlighted the F9 acetone fraction as the most cytotoxic. Inhibitory activity was dose-and time-dependent with an IC50 of 16.3 μg/mL after 48 h of treatment (Figure 2). The most active fraction (F9 acetone fraction) was then selected for further investigation.

*Apoptosis induction in MCF-7 cells by the F9 acetone fraction*

**Figure 3:**
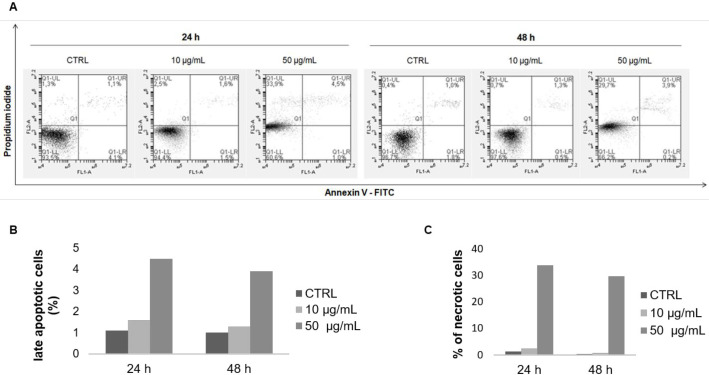
Annexin V-FITC/ PI double staining analysis of apoptosis in MCF-7 cells. Cells were treated with the F9 acetone fraction at 10 and 50 μg/mL for 24 and 48 h, and then stained with annexin V-FITC/PI and analyzed by flow cytometry. (A) Representative flow cytometry results of apoptosis: lower left quadrant, annexin V^-^/PI^-^, living cells; lower right quadrant, annexin V^+^/PI^-^, early apoptotic cells; upper right quadrant, annexin V^+^/PI^+^, late apoptotic cells; upper left quadrant, annexin V^−^/PI^+^, necrotic cells. Quantification of the percentage of (B) late apoptotic and (C) necrotic cells compared to the control group

**Figure 4:**
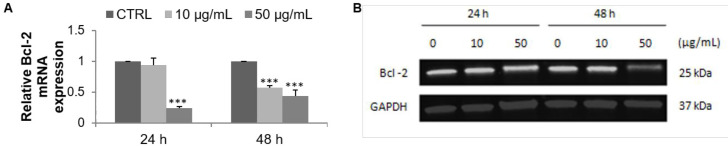
Effect of F9 acetone fraction on apoptosis in MCF-7 cells after 24 and 48 h of treatment at 10 and 50 μg/mL. (A) RT-qPCR analysis of Bcl-2 mRNA expression, the mRNA expression was normalized to 18S and the results are expressed relative to the control set at 1. (B) Western blot analysis of Bcl-2 protein level, GAPDH is used as a control

Apoptosis was first measured by flow cytometry after double staining with Annexin V-FITC and PI. F9 acetone fraction increased the percentage of late apoptotic cells in a dose- and time-dependent manner (Figure 3A), from 1.1% (CTRL) to 1.6% (10 µg/mL) and 4.5% (50 µg/mL) after 24 h, and from 1% (CTRL) to 1.3% (10 µg/mL) and 3.9% (50 µg/mL) after 48 h (Figure 3B). In addition, a marked increase of necrotic cells was observed after 24 and 48 h of exposure to 50 µg/mL from 1.3% (CTRL) to 33.9% and from 0.4% (CTRL) to 29.7%, respectively (Figure 3C). The expression profile of the anti-apoptotic marker Bcl-2 was then evaluated with RT-qPCR and Western blot. The mRNA expression of Bcl-2 in MCF-7 cells was noticeably decreased in a dose- and time-dependent manner after incubation with the F9 acetone fraction. As shown in Figure 4A, mRNA expression of Bcl-2 decreased with increasing concentration of the algal fraction from 0.9 to 0.2 and from 0.5 to 0.4 compared to the control defined as 1 after 24 and 48 h of incubation, respectively. 

Similarly, the expression level of Bcl-2 decreased in a dose- and time-dependent manner (Figure 4B). The bands were quantified and expressed as a percentage of the control group, concluding a marked decrease at 50 µg/mL of F9 acetone fraction from 83.3% at 24 h to 46.5% at 48 h (data not shown).

*Flotillin-2 expression reduction in MCF-7 cells by the F9 acetone fraction*

**Figure 5:**
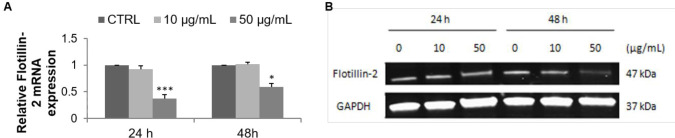
Effect of F9 acetone fraction on Flotillin-2 expression in untreated MCF-7 cells (CTRL) and in MCF-7 cells treated at 10 and 50 μg/mL for 24 and 48 h. (A) RT-qPCR analysis, the results were normalized relative to 18S ribosomal RNA level, expressed relative to untreated cells defined as 1.00. (B) Western blot representative, GAPDH was used as a control for protein loading

FLOT2 is a lipid raft marker which promotes the progression of several types of cancer. FLOT2 mRNA expression decreased in a dose- dependent manner from 0.92 (10 µg/mL) to 0.37 (50 µg/mL) and from 1.01 (10 µg/mL) to 0.59 (50 µg/mL) relative to the control after 24 and 48 h of treatment respectively (Figure 5A). Western blot pointed out that FLOT2 protein expression was reduced in F9 acetone fraction-treated MCF-7 cells, as evidenced by the decreased intensity of the targeting protein bands after 48 h of treatment with 50 µg/mL (Figure 5B).

## Discussion

Many publications have highlighted the potential implications of natural products of marine macroalgae that exhibit cytotoxic activity (10, 12). In this study, we have reported for the first time the cytotoxic activity of the CH2Cl2/MeOH (50/50, v/v) extract of the species *L. papillosa *collected from the Lebanese coast. To date, numerous studies have been conducted on the cytotoxic potential of various *L. papillosa* extracts on several human cancer cell lines. Especially the acetone extract of the Brazilian alga *L. papillosa* which showed a moderate cytotoxic activity on several cancer cell lines, including HL-60 (leukemia), B-16 (murine melanoma), HCT-8 (human colon carcinoma), MCF-7 (human breast carcinoma), and CEM (leukemia) cancer cell lines with an IC50 of 9.9, 13.9, 15.6, 31.6, and 49.0 μg/mL, respectively (31). In another study, no cytotoxic activity on the three breast cancer cell lines (MDA-MB-231, MCF-7 and T-47D) was detected for the ethanol extract of *L. papillosa* collected from the Iranian coast of the Persian Gulf after 72 h of incubation (32). However, the ethanol extract of *L. papillosa* from the Aegean Sea in Turkey inhibited the growth of the prostate (LNCa, PC-3) and breast (MCF-7) cancer cells at 50 μg/mL, but not of the non-tumorigenic epithelial cells (MCF-10A) (33).

Our results are consistent with those of Torres *et al.* but diverge from other studies (31-33). Variations in the cytotoxic effect of the same algal species can be explained by the choice of extraction solvent and its ability to recover bioactive compounds, as well as by seasonal and geographical fluctuations due to changes in environmental factors such as temperature, light, and salinity (13, 34). Regarding literature of *Laurencia*, several extraction solvents were used. A mixture of CH2Cl2/MeOH seems to be relevant, as it is the most frequent used and it demonstrated ability to extract a wide range of molecules. On the whole, algae collection (geography and season), extraction and treatment conditions were optimal since our *L. papillosa* extract was active compared to the previous studies.

Likewise, some metabolites such as aromatic compounds and fatty acid amide have been isolated from *L. papillosa* species and their cytotoxic potential against several cancer cell lines has been previously studied. The results showed that the aromatic compounds, 4-hydroxy-benzaldehyde, and 4-methoxy-benzyl alcohol were inactive against KB cancer cells, as well as the fatty acid amide, papillamide, which did not show any cytotoxic activity against P-388 (mouse leukemia) and B-16 (mouse melanoma) cancer cells after 96 h of incubation (IC50 greater than 100 μg/mL) (18, 20). However, most compounds isolated from *L. papillosa *have not been tested for cytotoxic activity on MCF-7 human breast cancer cells. Many other reports relate to the cytotoxic activity of the compounds isolated from the genus *Laurencia *against MCF-7 cells. For example, caespitol isolated from the Brazilian* L. catarinensis *and teuhetenone A from the Red Sea* L. obtusa *are both sesquiterpenes with high cytotoxic activity against MCF-7cells (IC50 of 9.7 and 22.8 µM respectively) (26, 35).

Besides, UPLC-HRMS-IT-TOF (Ultra Performance Liquid Chromatography coupled with High Resolution Mass Spectrometry Ion Trap Time of Flight) analyses were performed (data not shown). Dereplication suggested the absence of already known cytotoxic metabolites in the F9 acetone fraction.

A bio-guided fractionation led to the isolation of 24 fractions. Among them, the F9 acetone fraction exhibited an important cytotoxic activity on MCF-7 cells. The cell growth inhibition can be explained by the triggering of the apoptosis process. The induction of apoptotic cell death by the F9 acetone fraction was therefore studied. The percentage of late-apoptotic cells increased while the expression of the anti-apoptotic marker Bcl-2 decreased on mRNA and protein levels. In general, apoptosis allows the normal development of the organism and the maintenance of cellular homeostasis resulting from a balance between proliferation and cell death, and any deregulation leads to cancer development and progression. Its induction causes various morphological, molecular, and biochemical changes such as chromatin condensation, DNA fragmentation, activation or inactivation of members of the Bcl-2 family, activation of the caspases, release of the cytochrome c in the cytoplasm, exposure of phosphatidylserine to the extracellular membrane, which can be estimated using various assays (36). Our findings are in agreement with other studies showing the pro-apoptotic effect of various metabolites of algae of the genus *Laurencia*, here are some examples. B16F1 melanoma cells treated with laurinterol, a halogenated sesquiterpene isolated from *Laurencia okamurai*, exhibited DNA fragmentation, cell shrinkage, chromatin compaction, nuclear blebbing, and apoptotic bodies (37). In addition, the percentage of the cells in the sub-G1 phase, the activities of different caspases, the transcriptional activity of p53, and the expression level of phospho-p53 were increased. Overall, these results indicated that laurinterol induces apoptosis via p53 activation. According to Campos *et al.*, elatol which is a sesquiterpene isolated from *Laurencia microcladia*, was able to induce cell cycle arrest in the G1 and sub-G1 phases in B16F10 murine melanoma cells leading the cells to apoptosis (38). The results of various biological tests demonstrated that elatol increased the number of apoptotic cells as well as the expression of the pro-apoptotic proteins p53, Bak and caspase-9, and decreased the expression of the anti-apoptotic protein Bcl-xL.

There are numerous cellular signal transduction pathways controlling the processes associated with tumor progression, such as the regulation of cell proliferation and apoptosis that occur in lipid rafts (39). These lipid rafts are defined as micro-domains within the plasma membrane enriched in cholesterol and glycosphingolipids. They serve as signaling platforms in particular for growth factor receptors such as the epidermal growth factor receptor (40). FLOT2 isolated from these micro-domains is used as a lipid raft marker protein. Its increased expression has been observed in the cancer cells relative to the normal cells, suggesting its involvement in the development and progression of various cancers including breast cancer. In addition, it has been reported that its down-regulation is associated with inhibition of cancer cell proliferation, migration, and invasion (41, 42). FLOT2 is therefore a useful biomarker and a good pharmacological target in oncology. The present study is the first to investigate the FLOT2 expression level in MCF-7 cells treated with an algal fraction. Quantitative PCR and Western blot analyses performed in this study showed a decrease in lipid raft marker expression, thus demonstrating the importance of membrane rafts as a target for cancer treatment.

**Table 1.** Primers used for RT-qPCR analysis of gene expression

**Gene**	**Fsorward primer sequence (5'-3')**	**Reverse primer sequence (5'-3')**
18S	GATGCGGCGGCGTTATTCC	CTCCTGGTGGTGCCCTTCC
Bcl-2	GATTGTGGCCTTCTTTGAG	GTTCCACAAAGGCATCC
Flottilin-2	CAAGATTGCTGACTCTAAGC	GCACAACCTCAATCTCAATC

## Conclusion

The present study demonstrated the cytotoxic activity of CH2Cl2/MeOH (50/50, v/v) extract of *L. papillosa* on human breast cancer cells MCF-7. Biological evaluations have highlighted the great interest of the F9 acetone fraction: cell growth inhibition, apoptosis induction by increasing the percentage of late apoptotic cells and decreasing the expression of the anti-apoptotic member Bcl-2. Moreover, a decrease in the expression of FLOT2, which is generally over-expressed in cancer cells, has been observed. Preliminary dereplication studies did not identify already known compounds. Further studies are therefore needed for the chemical characterization of the metabolite associated with observed biological activities, suggesting that the red alga *L. papillosa* collected from the Lebanese coast could be a potential source of cytotoxic metabolites.
